# Caspase-1-Inhibitor AC-YVAD-CMK Inhibits Pyroptosis and Ameliorates Acute Kidney Injury in a Model of Sepsis

**DOI:** 10.1155/2021/6636621

**Published:** 2021-06-10

**Authors:** Mei Yang, Jin-tao Fang, Ni-shang Zhang, Long-jiang Qin, Yang-yang Zhuang, Wei-wei Wang, Hai-ping Zhu, Yan-jie Zhang, Peng Xia, Yan Zhang

**Affiliations:** ^1^Department of Intensive Care Unit, The First Affiliated Hospital of Wenzhou Medical University, Wenzhou, Zhejiang Province, China 325015; ^2^Transplantation Centre, The First Affiliated Hospital of Wenzhou Medical University, Wenzhou, Zhejiang Province, China 325015; ^3^Wenzhou Medical University, Wenzhou, Zhejiang Province, China 325015; ^4^Medical Engineering Department, The First Affiliated Hospital of Wenzhou Medical University, Wenzhou, Zhejiang Province, China 325015

## Abstract

**Objective:**

To observe the protective effect of AC-YVAD-CMK on sepsis-induced acute kidney injury in mice and to explore its possible mechanisms primarily.

**Methods:**

Eighteen male C57BL/6 mice were randomly divided into sham-operated group (Control), cecal ligation and puncture group (CLP), and CLP model treated with AC-YVAD-CMK group (AC-YVAD-CMK) (*n* = 6 in each group). Mice were sacrificed at 24 h after operation, and blood and kidney tissue samples were collected for analyses. Histologic changes were determined microscopically following HE staining. The expression of Ly-6B and CD68 was investigated using immunohistochemistry. Serum concentrations of creatinine (sCR) and blood urea nitrogen (BUN) were measured. Serum levels of interleukin-1*β* (IL-1*β*), interleukin-18 (IL-18), TNF-*α*, and interleukin-6 (IL-6) were determined by ELISA. The expressions of Caspas-1, NLRP-1, IL-1*β*, and IL-18 in renal tissues were investigated using Western blot. Immunofluorescence staining was used to detect the expression of GSDMD protein in renal tissues.

**Results:**

AC-YVAD-CMK treatment significantly alleviates sepsis-induced acute kidney injury, with decreased histological injury in renal tissues, suppresses the accumulation of neutrophils and macrophages in renal tissues, and decreased sCR and BUN level (*P* < 0.05). Attenuation of sepsis-induced acute kidney injury was due to the prohibited production of inflammatory cytokines and decrease expression of Caspas-1, NLRP-1, IL-1*β*, and IL-18 in renal tissues. In addition, AC-YVAD-CMK treatment significantly reduced the expression of GSDMD in renal tissues compared to those observed in controls (*P* < 0.05).

**Conclusions:**

We demonstrated a marked renoprotective effect of caspase-1-inhibitor AC-YVAD-CMK in a rat model of sepsis by inhibition of pyroptosis.

## 1. Introduction

Sepsis refers to a systemic inflammatory response caused by infection that can lead to shock, multiple organ dysfunction syndromes (MODS), and even death. Severe infection and septic shock are one of the most common causes of acute kidney injury (AKI), accounting for nearly 50% of cases of acute kidney failure (AKI) [1]. The incidence of AKI, which is directly proportional to the severity of infection, accounts for approximately 19% of patients with systemic infections, 23% of patients with severe infections, and 51% of patients with septic shock [2, 3].

Recently, the inflammasome signaling pathway has attracted widespread attention. As the central part of inflammatory response, inflammasomes are closely related to various diseases, such as immune inflammatory diseases, metabolic diseases, and nervous system diseases, especially playing a crucial role in infectious diseases [4].

NLRP1, the earliest reported inflammasome, is highly expressed in renal mononuclear phagocytes including dendritic cells and macrophages [5, 6] and is in a partially preassembled state. When activated, it can be further assembled and hydrolyze caspase-1 [7], thereby resulting in inflammation.

Besides, inflammasome also induces pyroptosis which is a new inflammatory cell death mode via the activation of caspase-1, triggering the possibility of repeated release and recognition of pathogenic bacteria [8]. NLRP1 inflammasome can directly bind to caspase-1 precursor and cause pyroptosis [9]. AC-YVAD-CMK, a well-known selective inhibitor of caspase-1, has been found to have a protective effect on the liver, brain, and lung in mouse models of septic shock in previous studies [10–12]; however, there are still no reports on whether AC-YVAD-CMK can prevent the kidney from damage in sepsis-induced acute kidney injury. Thus, our present study was performed to examine the effects of AC-YVAD-CMK on sepsis-induced acute kidney injury model.

Here, we examined whether sirolimus plays an important role in renal tubular apoptosis in the sepsis-induced acute kidney injury model.

## 2. Materials and Methods

### 2.1. Animals

Male C57BL/6 mice (6–8 weeks, weight 18–22 g) were purchased from Shanghai Laboratory Animal Co. (SLAC), Ltd. (Laboratory Animal Certificate: SCXK [HU] 2012-0002). The mice were housed in the Laboratory Animal Center of Wenzhou Medical University (license: SYXK (ZHE) 2010-0150) under standard temperature and humidity, light/dark cycles for 12 h and had free access to food and water. All animal-related experimental procedures were performed under the international guidelines for the care and use of laboratory animals and were approved by the Institutional Animal Care and Use Committee (IACUC) of Wenzhou Medical University (approval no: wydw2016-0079). The mice were allowed to acclimatize to the cages for 3 days before surgery.

### 2.2. CLP in Mice

The mice were randomly divided into three groups (*n* = 6 in each group): sham-operated group (Control), cecal ligation and puncture group (CLP), and CLP model treated with AC-YVAD-CMK group (AC-YVAD-CMK). Before surgery, the mice were anesthetized with an intraperitoneal (ip) injection of 80 mg/kg sodium pentobarbital (Merial, Hallbergmoos, Germany), and during surgery, they were placed on a heated table to maintain rectal temperature at 37–38°C. CLP-induced sepsis in mice was carried out as previously described [13]. Briefly, the mice underwent 1.5 cm midline laparotomy incision and fully exposed the cecum. The cecum was ligated at 1 cm away from the cecum tail with a 4-0 silk suture. A 20-gauge needle was used to perforate the blind end and squeezed little feces to induce sepsis. The cecum was returned to the peritoneal cavity and sutured the abdominal incision layer by layer. After the surgery, 1 ml of 0.9% sterile saline solution (37°C) was injected subcutaneously for liquid resuscitation. The sham group was operated as before but not cecal ligation and perforation. For the AC-YVAD-CMK group, CLP was performed, and the AC-YVAD-CMK (Sigma) contained saline (0.2 mg/ml, 37°C, 5 ml/100 g) was administered for resuscitation. All mice were sacrificed at 24 h after surgery, and the kidney and serum samples were harvested for analyses. After modeling, the rats developed fever (the temperature of the rectum was more than 1°C before modeling), the respiratory rate increased (doubled the breathing rate before modeling), the heart rate was increased (doubled the heart rate before modeling), and the oral and nasal secretions increased; malaise, vertical hair, less movement, etc., are regarded as a sign of successful model building.

### 2.3. Hematoxylin and Eosin Staining (H&E) Staining

The upper sections of renal tissue were routinely fixed in 4% paraformaldehyde for at least 6 hr and paraffin embedded. Tissue sections at 5 *μ*m were obtained, and the slides were deparaffinized with xylol and subsequently hydrated with gradient ethyl alcohol and running water. After washing, the sections were stained with hematoxylin for 3 min and eosin for 1 more min. The sections were subsequently visualized by optical microscopy at 200x magnification.

### 2.4. Renal Function Assessment

Blood samples from each mice were collected to measure the level of serum creatinine (Cr) and blood urea nitrogen (BUN) using an Olympus AU400 automated chemistry analyzer (Olympus, Tokyo, Japan).

### 2.5. Immunohistochemistry (IHC)

The expressions of Ly-6B (diluted 1 : 300; Rabbit Polyclonal Antibody, Cat No: YB-19701R, Ybscience, China) and CD68 (diluted 1 : 300; Rabbit Polyclonal Antibody, Cat No: AF6432, Sino Biological Inc, China) were assessed in paraffin-embedded tissue sections. Paraffin-embedded sections were dewaxed (or frozen sections were hydrated) and microwave oven heated in 0.1 M sodium citrate buffer for 15 min. After the serum block, sections were incubated with primary antibodies in PBS with 3% BSA overnight at 4°C. Sections were washed, and the primary antibodies were detected using the ABC method and developed with 3,3-diaminobenzidine (DAB) to produce a specific antigen brown color. The number of Ly-6B(+) and CD68(+) cells in the renal tissues were counted in four different fields, and mean was found by using the image analysis software “Leica Qwin.” Percentage of Ly-6B and CD68 positive cells was calculated on the number of Ly-6B-positive cells and CD68-positive cells out of 100 total cells that were counted.

### 2.6. Cytokine Analysis

Blood samples from each mice were collected by cardiac puncture and used for determining the concentration of IL-1*β*, IL-18, TNF-*α*, and IL-6 in serum using an enzyme-linked immunosorbent assay (ELISA) in accordance with the manufacturer's instructions (Westang, Shanghai, China).

### 2.7. Western Blotting

Western blotting was performed as previously described [14] to detect the expression of Caspas-1, NLRP-1, IL-1*β*, and IL-18 in the renal tissues. Kidney tissues were lysed in RIPA buffer, and the total protein concentrations were determined. Isolated proteins (20 *μ*g per specimen) were run on a 10% SDS-polyacrylamide electrophoresis gel and transferred onto nitrocellulose membranes (Hybond C Extra, Amershan Biosciences, Little Chalfon, USA). The membrane was incubated in a blocking buffer A (PBS, 5% nonfat milk, and 0.1% Tween-20) and incubated overnight at 4°C with primary rabbit anti-rat Caspas-1 (diluted 1 : 150; Rabbit Polyclonal Antibody, Cat No: ab138483, Abcam Biotechnology, Cambridge, MA), NLRP-1 (diluted 1 : 300; Rabbit Polyclonal Antibody, Cat No: ab36852, Abcam Biotechnology, Cambridge, MA), IL-1*β* (diluted 1 : 200; Rabbit monoclonal Antibody, Cat No: ab254360, Abcam Biotechnology, Cambridge, MA), IL-18 (diluted 1 : 200; Rabbit monoclonal Antibody, Cat No: ab207323, Abcam Biotechnology, Cambridge, MA), and *β*-actin (Abcam Biotechnology, Cambridge, MA). Then, the membrane was washed once for 15 min and twice for 5 min in PBS, followed by a peroxidase-conjugated sheep anti-rabbit IgG (Santa Cruz Biotechnology) at a 1 : 10000 dilution. At last, the signal was detected with an enhanced chemiluminescence kit (Westang, Shanghai, China) according to the manufacturer's instructions. We used a bioimage analysis system (Bio-Rad, USA) to analyze the bands.

### 2.8. Immunofluorescence

Immunofluorescence staining was performed using 4 *μ*m frozen sections to assess the pyroptosis in renal tissues. Sections were blocked with serum-free protein block (Dako, Carpinteria, CA) followed by incubation with primary antibodies, polyclonal rabbit anti-mouse GSDMD antibody (diluted 1 : 200; Rabbit monoclonal Antibody, Cat No: ab219800, Abcam Biotechnology, Cambridge, MA) for 1 h. Subsequently, the sections were incubated with Alexa-488 conjugated goat anti-rabbit antibody (1 : 100 dilution, A11008; Invitrogen, Carlsbad, CA) for 1 h. Sections were observed using a microscope equipped with a digital camera (Nikon Instruments, Melville, NY). Normal rabbit IgG was used as a negative control. Five glomeruli were sampled randomly from each specimen of each group at 400x magnification using the Image-pro plus 5.0 software (Media Cybernetics, USA), and mean fluorescence intensity (MFI) of glomeruli for each specimen was used to express the relative quantity of GSDMD protein.

### 2.9. Statistical Analysis

Data presented as means ± SEM, and one-way ANOVA test was used for statistics among the three groups. Differences were considered statistically significant if the *P* value was less than 0.05.

## 3. Result

### 3.1. AC-YVAD-CMK Alleviated Renal Injury of Mice after CLP

**Figure 1:**
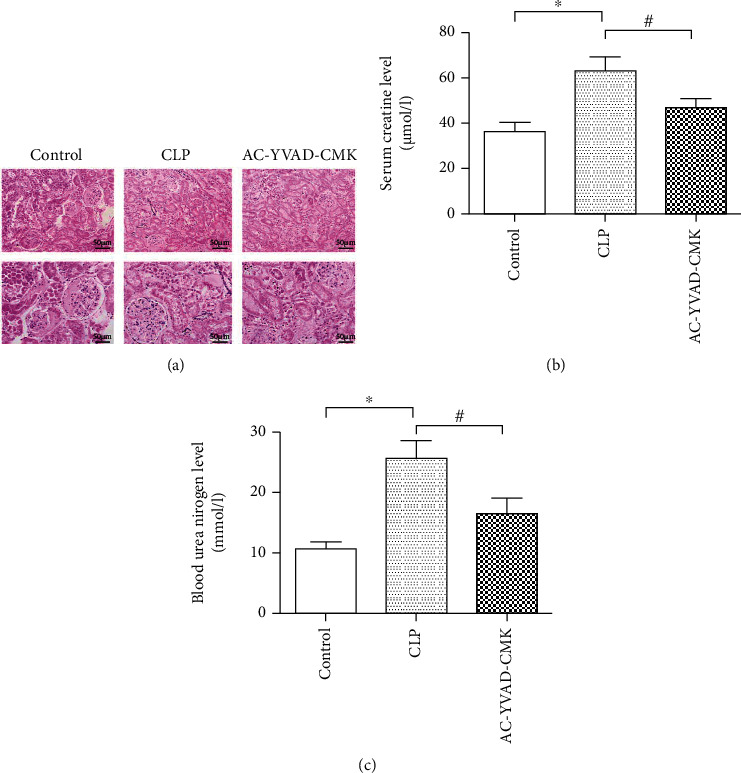
(a) AC-YVAD-CMK attenuated the histological changes in the renal tissues after cecal ligation and puncture. (b) AC-YVAD-CMK significantly reduced the level of serum creatinine after cecal ligation and puncture. (c) AC-YVAD-CMK significantly reduced the level of blood urea nitrogen after cecal ligation and puncture. ^∗^*P* < 0.05 in comparison with the Control group. ^#^*P* < 0.05 in comparison with the CLP group. *N* = 6 for each group. The bar represents mean ± s.e.m.

There was no obvious change in renal tissue in the Control group. In the CLP group, the glomerulus shrinks significantly, the kidney tissue is diffused with neutrophil infiltration, the interstitial edema is obvious, the balloon expands, and the tubular epithelium was partially cast. The cell vacuole degeneration and swelling, renal tubule lumen occlusion, renal tubule cavity expansion, and the cast are formed. The infiltration of monocyte-macrophage in the AC-YVAD-CMK treated group was less than that in the CLP group. The renal tubules showed focal damage, part of renal tubular epithelium was turbid and swollen, vacuolar degeneration, and interstitial edema was alleviated (Figure 1(a)).

### 3.2. AC-YVAD-CMK Improve Renal Function after CLP

Mice subjected to cecal ligation and puncture in the CLP group showed a significant increase in SCr and BUN levels compared with rats in the sham group (*P* < 0.05). The negative effects on renal function in response to cecal ligation and puncture were significantly reduced in the AC-YVAD-CMK treatment group compared with the CLP group, in relation to levels of SCr and BUN (*P* < 0.05) (Figures 1(b) and 1(c)).

### 3.3. AC-YVAD-CMK Suppresses the Accumulation of Neutrophils and Macrophages in Renal Tissues after CLP

**Figure 2:**
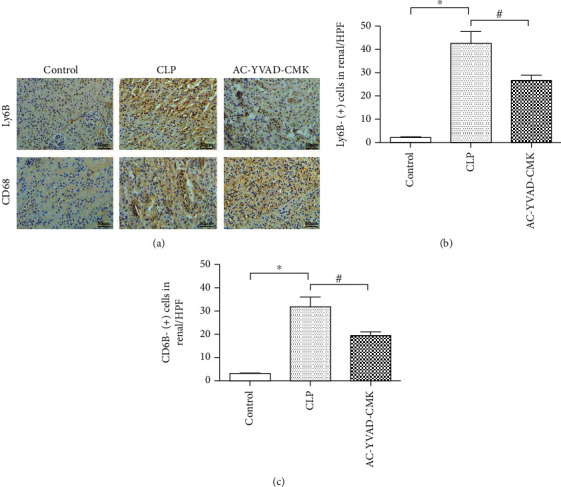
(a) AC-YVAD-CMK suppressed the accumulation of neutrophils and macrophages in renal tissues after CLP. (b) Comparison of the expression for Ly-6B-(+) cells in the renal tissues in each group. (c) Comparison of the expression for CD68-(+) cells in the renal tissues in each group. ^#^*P* < 0.05 in comparison with the CLP group. *N* = 6 for each group. The bar represents mean ± s.e.m.

To investigate whether the AC-YVAD-CMK treatment could suppress the accumulation of neutrophils and macrophages in renal tissues after CLP, we examined the changes of neutrophils and macrophages identified as Ly-6B-positive cells and CD68-positive cells (Figure 2(a)). Compared with the control group, Ly-6B-positive cells and CD68-positive cells in the CLP group were increased significantly (Figures 2(b) and 2(c)). In the AC-YVAD-CMK treatment group, Ly-6B-positive cells and CD68-positive cells were significantly decreased than those in the CLP group (Figures 2(b) and 2(c)) (*P* < 0.05).

### 3.4. AC-YVAD-CMK Suppresses Inflammatory Responses in Septic Mice

**Figure 3:**
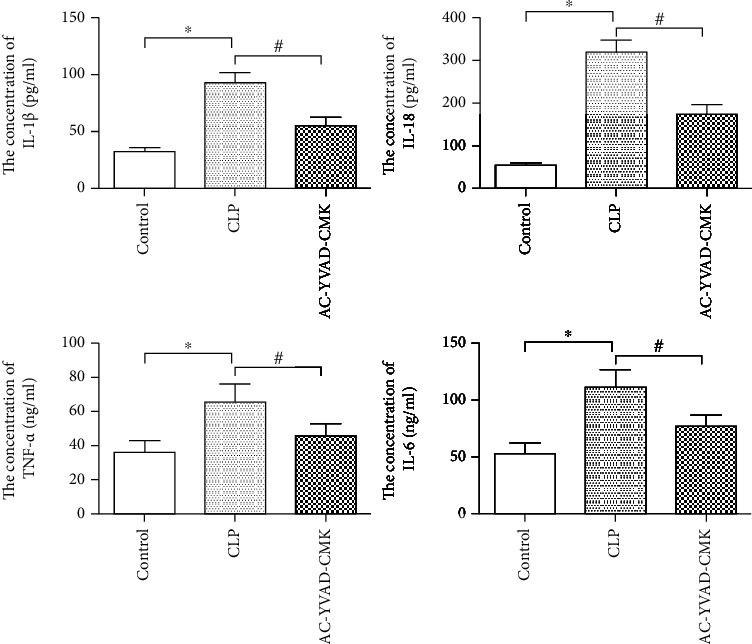
AC-YVAD-CMK significantly reduced the concentration of IL-1*β*, IL-18, TNF-*α*, and IL-6 after cecal ligation and puncture. ^∗^*P* < 0.05 in comparison with the Control group. ^#^*P* < 0.05 in comparison with the CLP group. *N* = 6 for each group. The bar represents mean ± s.e.m.

Mice in the CLP group showed strong upregulation of inflammatory cytokines (IL-1*β*, IL-18, TNF-*α*, and IL-6) compared with the Control group (*P* < 0.05) (Figure 3). All inflammatory cytokine concentrations were reduced in the AC-YVAD-CMK treated group compared with that in the CLP group (*P* < 0.05) (Figure 3).

### 3.5. AC-YVAD-CMK Inhibits Pyroptosis Induced by CLP

**Figure 4:**
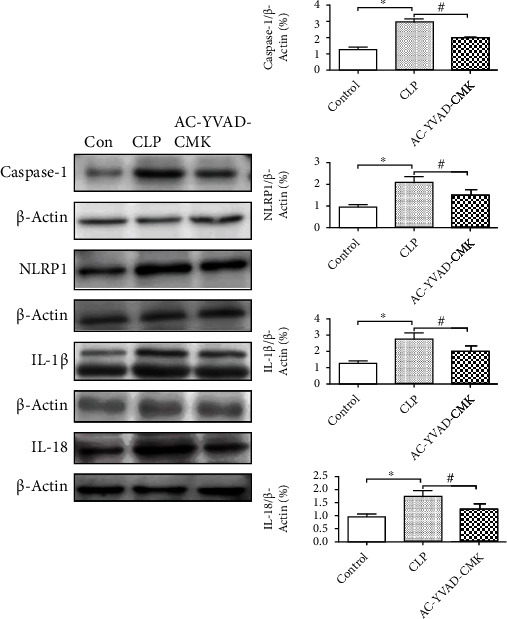
Western blot showed AC-YVAD-CMK significantly inhibits the expression of Caspas-1, NLRP-1, IL-1*β*, and IL-18 in the renal tissues after cecal ligation and puncture. The same blot was stripped and reprobed with actin to confirm equal loading. ^∗^*P* < 0.05 in comparison with the Control group. ^#^*P* < 0.05 in comparison with the CLP group. *N* = 6 for each group. The bar represents mean ± s.e.m.

**Figure 5:**
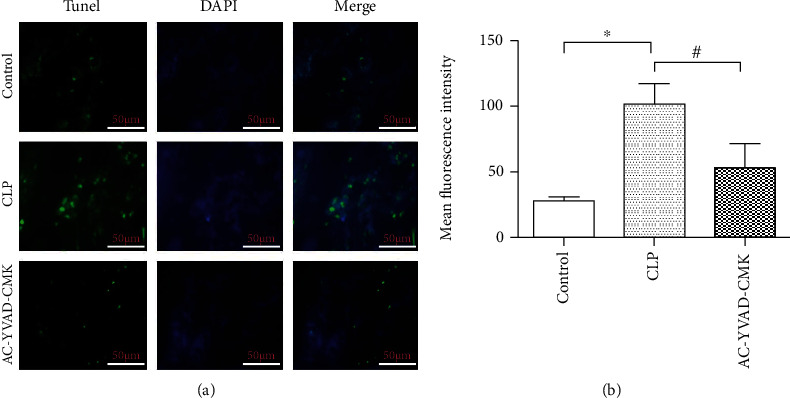
AC-YVAD-CMK suppresses expression of pyroptosis related protein GSDMD in the renal tissues after cecal ligation and puncture.

To explore whether AC-YVAD-CMK could affect the NLRP1 inflammasome signaling pathway to improve sepsis-induced acute kidney injury, we measured the expression levels of Caspas-1, NLRP-1, IL-1*β*, and IL-18 by Western blotting. The results showed that AC-YVAD-CMK treatment significantly reduced the expression levels of Caspas-1, NLRP-1, IL-1*β*, and IL-18 compared with those of the CLP group, confirming that AC-YVAD-CMK could reduce pyroptosis and sepsis-induced acute kidney injury (Figure 4). Quantification of the relative protein expression levels verified these findings (*P* < 0.05) (Figure 4). Additionally, immunofluorescence showed that the expression of pyroptosis related protein GSDMD in the CLP group were significantly increased compared with the sham group. AC-YVAD-CMK treatment significantly reduced the expression of GSDMD in renal tissues compared with that in the CLP group (Figure 5).

## 4. Discussion

In this study, we demonstrated that caspase-1-inhibitor AC-YVAD-CMK could significantly alleviated sepsis-induced acute kidney injury, with decreased histological injury in renal tissues, suppresses the accumulation of neutrophils and macrophages in renal tissues, and decreased sCR and BUN level. AC-YVAD-CMK treatment significantly decrease the serum levels of IL-1*β*, IL-18, IL-6, and TNF-*α*; decrease the protein expression of NLRP1, caspase-1, IL-1*β*, and IL-18in renal tissue; and inhibits pyroptosis induced by CLP. These results show that AC-YVAD-CMK could decrease the expression of NLRP1 inflammasome, inhibit the pyroptosis of renal tubular epithelial cells mediated by inflammasome pathway, and relieve the release of inflammatory factors IL-1*β* and IL-18 induced by NLRP1 inflammasome, showing a protective effect on sepsis-induced acute kidney injury.

Severe infection and its associated septic shock are considered to be one of the most common causes of acute kidney injury (AKI), accounting for about 50% of cases of acute kidney failure (AKI) [2]. The occurrence of AKI will promote and aggravate the damage of other organs, resulting in MODS and increasing mortality. The mortality rate of patients with severe infection complicated by acute renal failure (ARF) is as high as 70%, which is significantly higher than the mortality rate of ARF from other causes [15]. The pathogenesis of infectious AKI is multifactorial, involving changes in renal hemodynamics, renal perfusion and renal cell function, and damage in renal cell, as well as complex inflammation and immune network responses induced by endotoxin or endotoxin-like substances [16]. These different factors affect each other again. At present, there are still no clinical measures and methods that can effectively prevent and treat sepsis-induced AKI; therefore, it is necessary to further study the drug therapy.

Despite a great advance in the field of critical care medicine over these years, AKI induced by sepsis and septic shock is still a major problem. As a systemic inflammatory disease, sepsis-induced AKI has caused a heavy economic pressure on patients' family during the treatment process, and its 70% fatality rate is unacceptable [1].

Bagshaw et al. showed that the incidence of AKI in patients with systemic infection reached 42.1%, and the mortality rate of patients with RIFLE classification as risk, injury, and failure was 23.4%, 28.2%, and 36.8%, respectively [17]. It has also been reported that the fatality rate of patients with severe infection complicated by acute renal failure can be as high as 70% [2, 18]. The RIFLE classification developed by ADQI (Acute Dialysis Quality Initiative) and the AKI staging established by AKIN (Acute Kidney Injury Network) can well judge the prognosis of patients with different stage of AKI [19, 20]. Studies in recent years have found that even a slight increase in serum creatinine (26.4 pmol/l) will significantly increase the mortality of critically ill patients [21].

The occurrence of AKI will promote and aggravate the damage of other organs, resulting in MODS and increasing mortality. Now worldwide attention is paid to the early treatment of septic shock. Various treatment methods are emerging, such as early goal directed therapy, intensive blood-glucose control, stress hormone therapy, and application of activated protein C and cluster therapy [22]. However, for the treatment of infectious AKI, there are no clinical measures and methods that can effectively prevent AKI and shorten the natural course of disease except renal replacement therapy when renal failure occurs. Unfortunately, the debate about the choice and timing of kidney replacement still exists.

Recently, extensive attention is put on the inflammasome signaling pathway. As the center of inflammatory response, inflammatory bodies are closely related to various diseases, including immune inflammatory diseases, metabolic diseases, and nervous system diseases, especially playing a key role in infectious diseases [4].

The inflammasome is an intracellular polyprotein complex composed by nucleotide-binding and oligomerization domain-like receptors (NLRs), cysteine-containing aspartate proteolytic enzyme 1 (caspase-1), and apoptosis-associated speck-like protein containing a CARD (ASC), which molecular weight about 700 KD [23, 24]. NLRs, as important classes of pattern recognition receptors of the immune system, are a large family of receptors in cells that can nonspecifically recognize microorganisms, endogenous stress response, and danger signals [15]. With the deepening of research, more types of inflammasome have been found. And even more studies have confirmed that the abnormal activation and regulation of inflammasome are closely related to many autoimmune inflammatory diseases, metabolic diseases, and kidney diseases. Twenty-three NLR family proteins have been found in humans and thirty-five in mice. The members of this family, such as NLRP1 (NALP1), NLRP3, NLRC4, NAIP5, NLRP6, and NLRP12, can participate in the assembly of inflammasome. As a member of the NLRs family, NLRP1 interacts with downstream caspase-1 to form the NLRP1 inflammasome [24, 25].

NLRP1 is the earliest reported inflammasome. Most of the NLRP1 contain a C-terminal CARD domain which can directly bind to the caspase-1 precursor without the participation of ASC through CARD-CARD isotype interaction. However, the participation of the ASC can significantly upregulate the activity of the NLRP1 inflammasome [25, 26]. NLRP1 is highly expressed in renal mononuclear phagocytes including dendritic cells and macrophages [5, 6] and is in a partially preassembled state. When activated, it can be further assembled and hydrolyze caspase-1 [7, 27], thereby resulting in inflammation. The specific activation mechanism of NLRP1 has not yet been fully elucidated. The mainstream activation pathway is that extracellular ATP may be involved in the activation of inflammasome, which binds to the P2X7 receptor of the ion channel to trigger potassium ion outflow [27]. Further research also showed that high extracellular potassium ion concentration activates NLRP1 inflammasome in neurons and increases the expression of caspase-1 in astrocytes [28]. The inflammasome is an intracellular polyprotein complex composed by NOD-like receptor (NLR) family, apoptosis-associated speck-like protein containing a CARD (ASC), and cysteine-containing aspartate proteolytic enzyme 1 (caspase-1).

When an infection or intracellular danger signal appears, the inflammasome recruits and activates caspase-1 precursor directly or through ASC to form activated caspase-1. The latter further promotes the cleavage and conversion of IL-1*β* and IL-18 and other inflammatory factors into a mature activated state to be released outside the cell to play a biological role [29].

Besides, inflammasome also induces pyroptosis which is a new inflammatory cell death method via the activation of caspase-1, triggering the possibility of repeated release and recognition of pathogenic bacteria [8]. Pyroptosis, a form of programmed cell death discovered in recent years, is characterized by dependence on caspase-1 and/or caspase-11, accompanied by early rupture of the plasma membrane and the release of a large number of proinflammatory factors [9]. As a way of cell death accompanied by inflammatory reaction, pyroptosis is no less important than apoptosis. In some pathological processes, pyroptosis is even more practical than apoptosis. It has been found that cell pyroptosis is widely involved in the occurrence and development of infectious diseases, nervous system related diseases, and atherosclerotic diseases.

The NLRP1 inflammasome can directly bind to the caspase-1 precursor, causing pyroptosis [30]. Pyroptosis have the characteristics of apoptosis and necrosis at the same time. Very similar to the morphology of apoptosis, pyroptosis exhibits nuclear condensation, chromatin DNA breakage, and positive TUNEL staining. In early cell death research, the cells showed positive TUNEL staining; the researchers misunderstood that the cell death was apoptosis. However, unlike apoptosis, during pyroptosis, the loss of cell membrane integrity leads to the release of cellular contents, inducing an inflammatory response. When pyroptosis occurs, small pores of 1-2 nm are formed in the cell membrane. It is these holes that disrupt the ion balance in the cell, allowing water to flow in, and then the cell swelling causes the membrane to rupture and ultimately results in cellular lysis. In different animal models, the suppression of inflammasome could alleviate the innate immune response, renal tubular epithelial cell death, and kidney damage [31]. Although there are few studies on the NLRP1 inflammasome, many results have confirmed its important role in the pathogenesis of kidney disease [32].

Pyroptosis has recently become a hot topic in kidney disease research [33], which is involved in the process of various kidney injuries [34, 35]. In chronic glomerulonephritis [29], diabetic nephropathy [36], and HIV-associated nephropathy [37], pyroptosis in renal tubular epithelial cells exists, and inhibiting pyroptosis in podocyte can reduce its damage [38]. In the process of renal ischemia-reperfusion injury, pyroptosis of renal tubular epithelial cells can also be observed [39]. Targeted blocking of pyroptosis of renal tubular epithelial cells can alleviate tubular damage caused by ischemia-reperfusion [40]. In the process of chronic renal interstitial fibrosis, pyroptosis promotes the inflammatory response of the kidney and the process of renal tubular epithelial to mesenchymal transition [41]. These studies all indicate that external damage signals have initiated the pyroptosis of renal tubular epithelial cells and podocytes, promoted the maturation and release of inflammatory factors, and aggravated kidney injury, while inhibiting pyrolysis can reduce the process of kidney injury.

Evidence from increasing animal studies shows that the histological feature of AKI in sepsis is the loss of renal tubular epithelial cells [42]. However, the exact molecular mechanism is not yet clear.

In this study, we found that pyroptosis related protein GSDMD in renal tubular epithelial cells was significantly increased during sepsis-induced AKI, suggesting that pyroptosis of renal tubular epithelial cells may be the key reason for the loss of renal tubular epithelial cell during AKI. The use of AC-YVAD-CMK, a caspase-1 inhibitor, could decrease the expression of NLRP1 inflammasome and attenuate the inflammasome-induced pyroptosis of renal tubular epithelial cells, showing an excellent renal protection.

All in all, AC-YVAD-CMK has a significant protective effect on sepsis-induced acute kidney injury in rats, thus providing a theoretical basis for its clinical application in the therapy of patients with sepsis-induced acute kidney injury. The mechanism may be related to blocking the expression of NLRP1 inflammasome, reducing the inflammasome-induced pyroptosis of renal tubular epithelial cells, increasing the activity of antioxidant enzymes and attenuating the oxidation products. Since all the current treatment experience comes from animal experiments, the mechanism and safety of AC-YVAD-CMK in the clinic still need to be further studied.

## Data Availability

The data used to support the findings of this study are included within the article.

## Supplementary Materials

Supplementary Materials Table 1: densitometry readings/intensity ratio of each band in each group. Figure 1: the expression of Caspas-1 in renal tissues after CLP. Figure 2: the expression of NLRP-1 in renal tissues after CLP. Figure 3: the expression of IL-1*β* in renal tissues after CLP. Figure 4: the expression of IL-18 in renal tissues after CLP.

## References

[1] Heresi G. A. (2004). Acute renal failure and sepsis. *The New England Journal of Medicine*.

[2] Remuzzi G., Horton R. (2013). Acute renal failure: an unacceptable death sentence globally. *Lancet*.

[3] Sun D., Aikawa N. (1999). The natural history of the systemic inflammatory response syndrome and the evaluation of SIRS criteria as a predictor of severity in patients hospitalized through emergency services. *The Keio Journal of Medicine*.

[4] Shao B. Z., Fatahian A., Bandepour M., Talebian A., Moosavi G. (2015). NLRP3 inflammasome and its inhibitors: a review. *Frontiers in Pharmacology*.

[5] Masood H., Che R. C., Zhang A. H. (2015). Inflammasomes in the pathophysiology of kidney diseases. *Kidney Diseases*.

[6] Lichtnekert J., Kulkarni O. P., Mulay S. R. (2011). Anti-GBM glomerulonephritis involves IL-1 but is independent of NLRP3/ASC inflammasome-mediated activation of caspase-1. *PLoS One*.

[7] Timoshanko J. R., Kitching A. R., Iwakura Y., Holdsworth S. R., Tipping P. G. (2004). Leukocyte-derived interleukin-1beta interacts with renal interleukin-1 receptor I to promote renal tumor necrosis factor and glomerular injury in murine crescentic glomerulonephritis. *The American Journal of Pathology*.

[8] Conforti-Andreoni C., Ricciardi-Castagnoli P., Mortellaro A. (2011). The inflammasomes in health and disease: from genetics to molecular mechanisms of autoinflammation and beyond. *Cellular & Molecular Immunology*.

[9] Bergsbaken T., Fink S. L., Cookson B. T. (2009). Pyroptosis: host cell death and inflammation. *Nature Reviews. Microbiology*.

[10] Wu D.-D., Pan P. H., Liu B. (2015). Inhibition of alveolar macrophage pyroptosis reduces lipopolysaccharide-induced acute lung injury in mice. *Chinese Medical Journal*.

[11] Fu Q., Wu J., Zhou X. Y. (2019). NLRP3/caspase-1 pathway-induced pyroptosis mediated cognitive deficits in a mouse model of sepsis-associated encephalopathy. *Inflammation*.

[12] Chen Y. L., Xu G., Liang X. (2016). Inhibition of hepatic cells pyroptosis attenuates CLP-induced acute liver injury. *American Journal of Translational Research*.

[13] Xu S., Gao Y., Zhang Q. (2016). SIRT1/3 activation by resveratrol attenuates acute kidney injury in a septic rat model. *Oxidative Medicine and Cellular Longevity*.

[14] Yang M., Zhuang Y. Y., Wang W. W. (2018). Role of Sirolimus in renal tubular apoptosis in response to unilateral ureteral obstruction. *International Journal of Medical Sciences*.

[15] Rangel-Frausto M. S., Pittet D., Costigan M., Hwang T., Davis C. S., Wenzel R. P. (1995). The natural history of the systemic inflammatory response syndrome (SIRS). A prospective study. *JAMA*.

[16] Rabb H. (2006). Immune modulation of acute kidney injury. *Journal of the American Society of Nephrology*.

[17] Cruz M. G., Dantas J. G., Levi T. M. (2014). Septic versus non-septic acute kidney injury in critically ill patients: characteristics and clinical outcomes. *Revista Brasileira de terapia intensiva*.

[18] Srisawat N., Lawsin L., Uchino S., Bellomo R., Kellum J. A., the BEST Kidney Investigators (2010). Cost of acute renal replacement therapy in the intensive care unit: results from The Beginning and Ending Supportive Therapy for the Kidney (BEST Kidney) study. *Critical Care*.

[19] Bagshaw S. M., George C., Bellomo R., the ANZICS Database Management Committee (2008). Early acute kidney injury and sepsis: a multicentre evaluation. *Critical Care*.

[20] Van Biesen W., Yegenaga I., Vanholder R. (2005). Relationship between fluid status and its management on acute renal failure (ARF) in intensive care unit (ICU) patients with sepsis: a prospective analysis. *Journal of Nephrology*.

[21] Chertow G. M., Soroko S. H., Paganini E. P. (2006). Mortality after acute renal failure: models for prognostic stratification and risk adjustment. *Kidney International*.

[22] Dellinger R. P., Levy M. M., Rhodes A. (2013). Surviving Sepsis Campaign: international guidelines for management of severe sepsis and septic shock, 2012. *Intensive Care Medicine*.

[23] Franchi L., Eigenbrod T., Muñoz-Planillo R., Nuñez G. (2009). The inflammasome: a caspase-1-activation platform that regulates immune responses and disease pathogenesis. *Nature Immunology*.

[24] Lechtenberg B. C., Mace P. D., Riedl S. J. (2014). Structural mechanisms in NLR inflammasome signaling. *Current Opinion in Structural Biology*.

[25] Lamkanfi M., Dixit V. M. (2014). Mechanisms and functions of inflammasomes. *Cell*.

[26] Schroder K., Tschopp J. (2010). The inflammasomes. *Cell*.

[27] Pelegrin P., Surprenant A. (2006). Pannexin-1 mediates large pore formation and interleukin-1beta release by the ATP-gated P2X7 receptor. *The EMBO Journal*.

[28] Silverman W. R., de Rivero Vaccari J. P., Locovei S. (2009). The pannexin 1 channel activates the inflammasome in neurons and astrocytes. *The Journal of Biological Chemistry*.

[29] Conley S. M., Abais J. M., Boini K. M., Li P. L. (2017). Inflammasome activation in chronic glomerular diseases. *Current Drug Targets*.

[30] Masters S. L., Gerlic M., Metcalf D. (2012). NLRP1 inflammasome activation induces pyroptosis of hematopoietic progenitor cells. *Immunity*.

[31] Chen K., Zhang J., Zhang W. (2013). ATP-P2X4 signaling mediates NLRP3 inflammasome activation: a novel pathway of diabetic nephropathy. *The International Journal of Biochemistry & Cell Biology*.

[32] Darisipudi M. N., Knauf F. (2016). An update on the role of the inflammasomes in the pathogenesis of kidney diseases. *Pediatric Nephrology*.

[33] Krautwald S., Linkermann A. (2014). The fire within: pyroptosis in the kidney. *American Journal of Physiology. Renal Physiology*.

[34] Linkermann A., Chen G., Dong G., Kunzendorf U., Krautwald S., Dong Z. (2014). Regulated cell death in AKI. *Journal of the American Society of Nephrology*.

[35] Kers J., Leemans J. C., Linkermann A. (2016). An overview of pathways of regulated necrosis in acute kidney injury. *Seminars in Nephrology*.

[36] Lin C. F., Kuo Y. T., Chen T. Y., Chien C. T. (2016). Quercetin-rich guava (Psidium guajava) juice in combination with trehalose reduces autophagy, apoptosis and pyroptosis formation in the kidney and pancreas of type II diabetic rats. *Molecules*.

[37] Haque S., Lan X., Wen H. (2016). HIV promotes NLRP3 inflammasome complex activation in murine HIV-associated nephropathy. *The American Journal of Pathology*.

[38] Thomasova D., Bruns H. A., Kretschmer V. (2015). Murine double minute-2 prevents p53-overactivation-related cell death (podoptosis) of podocytes. *Journal of the American Society of Nephrology*.

[39] Yang J. R., Yao F. H., Zhang J. G. (2014). Ischemia-reperfusion induces renal tubule pyroptosis via the CHOP-caspase-11 pathway. *American Journal of Physiology. Renal Physiology*.

[40] Wu H., Huang T., Ying L. (2016). MiR-155 is involved in renal ischemia-reperfusion injury via direct targeting of Fox O3a and regulating renal tubular cell pyroptosis. *Cellular Physiology and Biochemistry*.

[41] Lorenz G., Darisipudi M. N., Anders H. J. (2014). Canonical and non-canonical effects of the NLRP3 inflammasome in kidney inflammation and fibrosis. *Nephrology, Dialysis, Transplantation*.

[42] Kaushal G. P., Basnakian A. G., Shah S. V. (2004). Apoptotic pathways in ischemic acute renal failure. *Kidney International*.

