# Supplementary material for: Caspase-1-Inhibitor AC-YVAD-CMK Inhibits Pyroptosis and Ameliorates Acute Kidney Injury in a Model of Sepsis
Source: Biomed Res Int. 2021 Jun 10;2021:6636621. doi: 10.1155/2021/6636621 (PMC8213477; doi:10.1155/2021/6636621)
Supplement: Supplementary Materials — Table 1: densitometry readings/intensity ratio of each band in each group. Figure 1: the expression of Caspas-1 in renal tissues after CLP. Figure 2: the expression of NLRP-1 in renal tissues after CLP. Figure 3: the expression of IL-1β in renal tissues after CLP. Figure 4: the expression of IL-18 in renal tissues after CLP. [file 6636621.f1.docx]

Dear editor:

In this supplemental materials, we add the densitometry readings/intensity ratio of each band and the whole blot (uncropped blots) showing all the bands

Table 1 densitometry readings/intensity ratio of each band in each group

|  | control | clp | ac-mk | control | clp | ac-mk | control | clp | ac-mk |
| --- | --- | --- | --- | --- | --- | --- | --- | --- | --- |
| caspas1 | 41829 | 90291 | 67567 | 37289 | 91886 | 66583 | 40479 | 89740 | 68259 |
| Actin | 32843 | 32092 | 36857 | 30886 | 31067 | 30996 | 32305 | 31006 | 38269 |
| caspas1/actin | 1.273605 | 2.813505 | 1.83322 | 1.207311 | 2.957672 | 2.148116 | 1.253026 | 2.894279 | 1.783663 |
| NLRP1 | 61676 | 114164 | 81798 | 64665 | 123256 | 84903 | 63991 | 110873 | 82198 |
| Actin | 53243 | 53345 | 52115 | 56098 | 55670 | 56032 | 55387 | 54889 | 53385 |
| NLRP1/actin | 1.158387 | 2.140107 | 1.569567 | 1.152715 | 2.214047 | 1.515259 | 1.155343 | 2.019949 | 1.539721 |
| IL-1B | 96907 | 145260 | 123838 | 94032 | 148779 | 119838 | 99766 | 152839 | 122761 |
| Actin | 58680 | 54576 | 56733 | 57755 | 55302 | 55863 | 60750 | 59632 | 58875 |
| IL1B/actin | 1.651449 | 2.661609 | 2.182821 | 1.628119 | 2.690301 | 2.145212 | 1.642239 | 2.563037 | 2.085113 |
| IL18 | 71466 | 125804 | 78986 | 66399 | 118934 | 76756 | 70736 | 120679 | 83663 |
| Actin | 74892 | 75211 | 67738 | 68254 | 67879 | 68325 | 72275 | 71321 | 72306 |
| IL18/actin | 0.954254 | 1.672681 | 1.166052 | 0.972822 | 1.752147 | 1.123396 | 0.978706 | 1.692054 | 1.157069 |

Figure-1. The expression of Caspas-1in renal tissues after CLP

Figure-2. The expression of NLRP-1in renal tissues after CLP

Figure-3. The expression of IL-1β in renal tissues after CLP

Figure-4. The expression of IL-18 in renal tissues after CLP
